# Calorimetric Investigation of Hydrogen-Atom Sublattice Transitions in the Ice VI/XV/XIX Trio

**DOI:** 10.1021/acs.jpcb.1c07508

**Published:** 2021-10-14

**Authors:** Tobias M. Gasser, Alexander V. Thoeny, Victoria Greussing, Thomas Loerting

**Affiliations:** Institute of Physical Chemistry, University of Innsbruck, 6020 Innsbruck, Austria

## Abstract

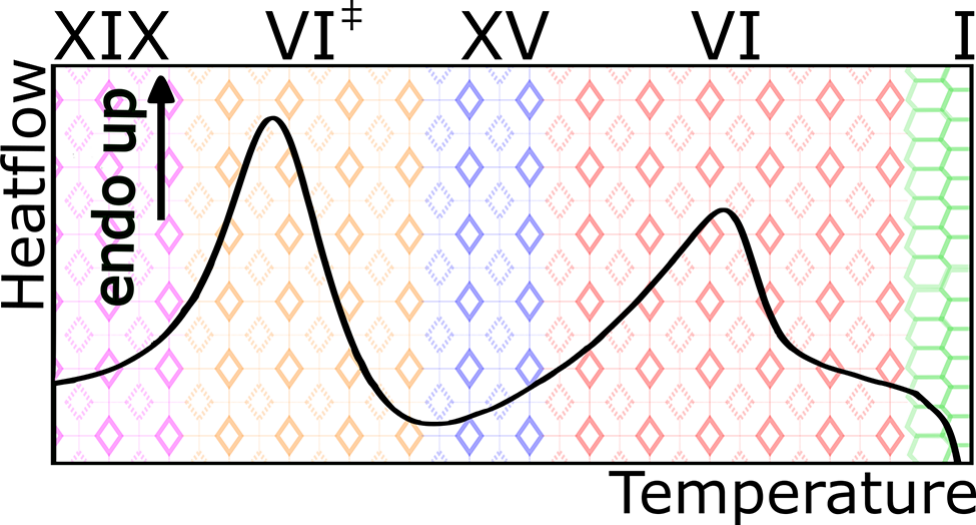

Ice XIX represents the latest discovery of ice polymorphs and exists in the medium pressure range near 1–2 GPa. Ice XIX is a partially hydrogen-ordered phase, by contrast to its disordered mother phase ice VI, which shares the same oxygen-atom network with ice XIX. Ice XIX differs in terms of the ordering of the hydrogen-atom sublattice, and hence the space group, from its hydrogen-ordered sibling ice XV, which also features the same type of oxygen network. Together, ice VI, XV, and XIX form the only known trio of ice polymorphs, where polymorphic transformations from order to order, order to disorder, and disorder to order are possible, which also compete with each other depending on the thermodynamic path taken and the cooling/heating rates employed. These transitions in the H-sublattice have barely been investigated, so we study here the unique triangular relation in the ice VI/XV/XIX trio based on calorimetry experiments. We reveal the following key features for H-sublattice transitions: (i) upon cooling ice VI, domains of ice XV and XIX develop simultaneously, where pure ice XV forms at ≤0.85 GPa and pure ice XIX forms at ≥1.60 GPa, (ii) ice XIX transforms into ice XV via a transient disordered state, (iii) ice XV recooled at ambient pressure features a complex domain structure, possibly containing an unknown H-ordered polymorph, (iv) recooled ice XV partly transforms back into ice XIX at 1.80 GPa, and (v) partial deuteration slows down domain reordering strongly. These findings not only are of interest in understanding possible hydrogen-ordering and -disordering processes in the interior of icy moons and planets but, more importantly, also provide a challenging benchmark for our understanding and parameterizing many-body interactions in H-bonded networks.

## Introduction

The family of ice polymorphs has recently grown through the discovery of ice XIX.^1,2^ Ice XIX is a partly hydrogen (H)-ordered phase related to its disordered counterpart ice VI. The structural model in the tetragonal space group 4̅ matches the neutron diffraction data best, both under pressure^2^ and after recovery to ambient pressure.^1^ Ice XIX is antiferroelectric, just like its triclinic *P*1̅ sibling ice XV.^3^ This makes the unique case for a “trio” of ice polymorphs sharing the same topological network of oxygen (O) atoms. Other ice polymorphs appear as “order–disorder duos” or “singles”, yet awaiting the discovery of their counterpart. For this reason, ice VI/XV/XIX represents a unique case that allows for the study of the triangular relationship between one disordered parental phase and two ordered children. In particular, the order-to-order transition from ice XIX to XV at ambient pressure is unprecedented in ice chemistry.^1,4^ Please note that both ice XIX and XV are partly ordered, where the phase transition between them results in a change of space group symmetry. Our notion of order follows the tradition in ice chemistry that has begun in the 1970s with the report of the first (partly) ordered ice phase, ice XI.^5,6^ That is, the order-to-order transition described here reflects a transition from one H-ordered space group to another, where the order is partial both before and after the transition. The criterion for the transition from one type of H-order to another type of H-order is the change of symmetry elements within the unit cell induced through H-atom rearrangement. Already years before the discovery of ice XIX, Shephard and Salzmann had noticed that the disordering transition from ice XV to VI shows features^7^ unknown for any other ordered ice polymorph. Rosu-Finsen et al. attributed the complexity to a disordered, deep glassy H-atom network below 129 K.^8,9^ By contrast, our group has advocated the existence of a novel H-ordered ice polymorph,^4,10^ now known as ice XIX. In a very recent work, Salzmann et al. claimed that “ice XIX” is H-disordered and originates from ice VI through lattice distortion.^11^ As pointed out by Hansen in an accompanying comment, it is unlikely though, “we are really looking at the same ice phase” because their preparation procedures are different.^12^

Not only in experiments but also from a theoretical viewpoint, the ordering of ice VI represents a challenge.^13,14^ The ice XIX unit cell has double the volume of the ice VI and XV unit cells, making supercells a necessity to be considered in theory work. Nanda and Beran have asked about the governing factors for the order in ice VI.^15^ Concerning the ice VI unit cell, Komatsu et al. found that all ordered configurations are almost degenerate.^16^ Because of the near-degeneracy, it seems conceivable that ice VI has a multitude of reaction paths upon cooling, possibly leading to a range of differently ordered domains within the ice VI matrix. For instance, a *Cc* phase has been considered in theory work but not discovered in the experiment.^13^ In this experimental work, we take a close look at pressure as a governing factor for the type of H-order that develops upon cooling ice VI. Furthermore, we examine whether it is possible to directly switch between the two ordered low-temperature phases ice XV and XIX, without taking the detour via the high-temperature form ice VI. As our main analytical tool, we use low-temperature differential scanning calorimetry, where subtle changes in heat flow indicate (endothermic) disordering and (exothermic) ordering transitions in the H-sublattice of the ice.

## Methods

### Sample Preparation

**Figure 1:**
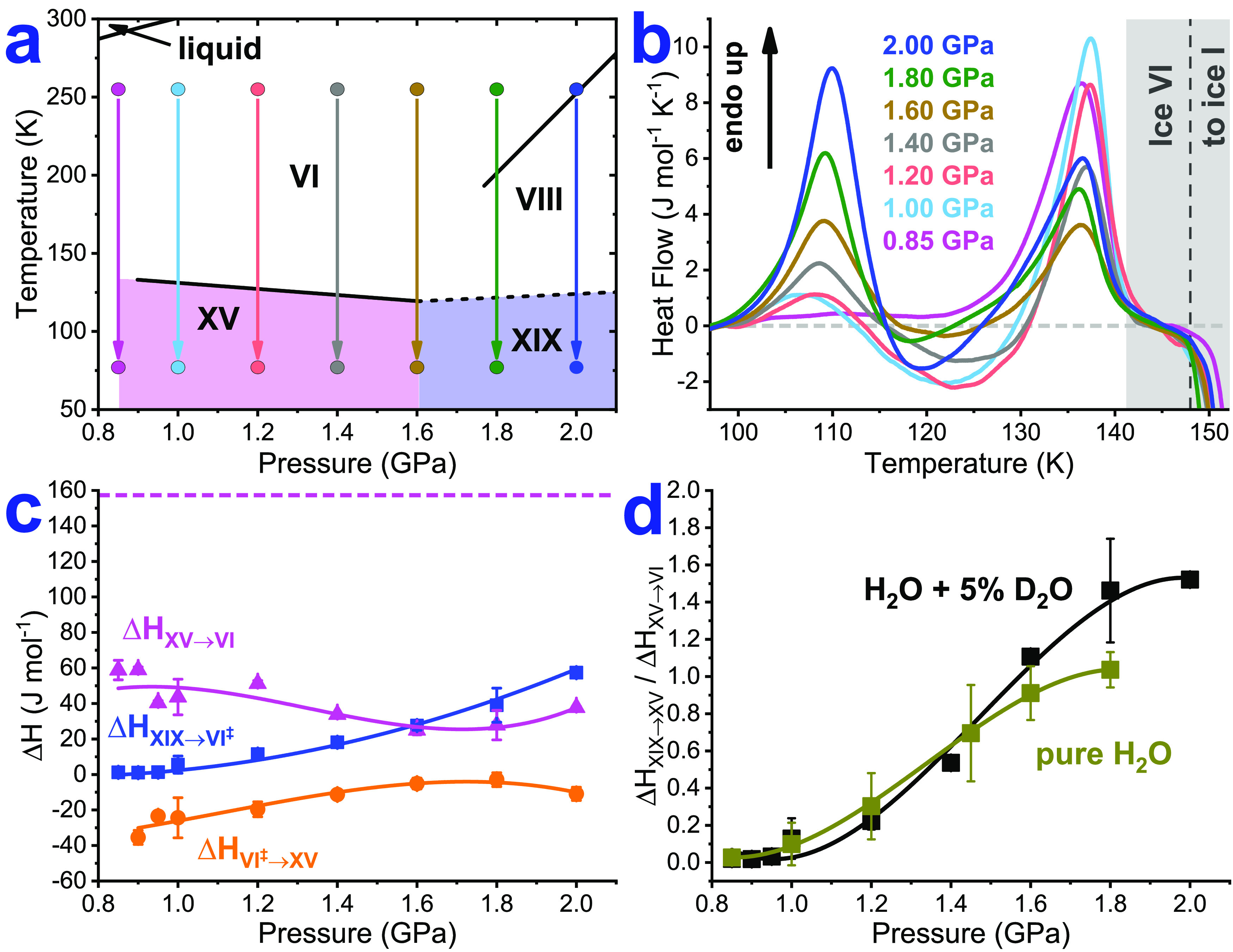
First set of experiments: (a) Schematic representation of sample preparation starting from 95% H_2_O and 5% D_2_O ice VI, containing 0.01 M HCl as a dopant. The phase diagram as shown by Yamane et al.^2^ (b) Calorimetry traces recorded upon heating recovered samples at 10 K min^–1^ at ambient pressure. The dashed gray line represents the baseline. (c) Transformation enthalpies for the ice XIX endotherm (blue), ice VI^‡^ exotherm (orange), and ice XV endotherm (purple). The dashed purple line marks the transformation enthalpy of recooled ice XV (wine red trace in Figure 3a). (d) Ratio of the areas for the ice XIX and XV endotherms for HCl-doped H_2_O samples (dark yellow trace) and samples containing 5% D_2_O (black trace). Lines in (c) and (d) are guides to the eye.

In general, the sample preparation follows the procedures outlined in our previous work on ice β-XV^4,10^ and XIX.^1^ In the following, we briefly describe the specific details such as sample volume, isotopic composition, dopant, and the thermodynamic path. Cooling rates represent a kinetic aspect and determine the time provided in the experiment for the H-atom sublattice to rearrange. For the data shown in Figure 1, ice XV/XIX was prepared at different pressures (0.85, 0.90, 0.95, 1.00, 1.20, 1.40, 1.60, 1.80, and 2.00 GPa) using a piston–cylinder setup and a ZWICK BZ100/TL3S universal testing machine. Six hundred microliters of the sample solution, composed of 95% H_2_O and 5% D_2_O and containing 0.01 M HCl as a dopant, were pipetted into an indium container and placed inside the 8 mm diameter bore of the cylinder. The role of the indium container is to reduce friction between the sample and the piston–cylinder at cryogenic temperatures, as originally proposed by Mishima et al.^17^ The sample was then (i) cooled to 77 K, (ii) compressed to one of the pressures specified above, (iii) heated to 255 K, (iv) cooled to 77 K, (v) decompressed, and (vi) recovered under liquid nitrogen. In step (iv), the samples were cooled from 160 to 90 K at a rate of 3 K min^–1^. Above 160 K and below 90 K, cooling rates of up to 20 K min^–1^ were applied. As a reference, 0.01 M HCl in H_2_O samples, without any added D_2_O, were prepared at pressures of 0.85, 1.00, 1.20, 1.45, 1.60, and 1.80 GPa.

**Figure 2:**
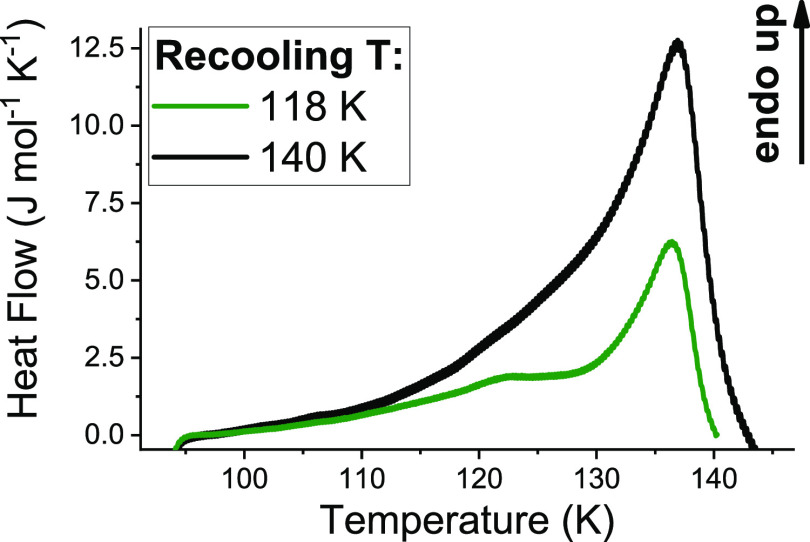
Second set of experiments. Calorimetry traces of recooled ice XV: ice XIX was transformed to recooled ice XV by either heating to 118 K and recooling at 1 K min^–1^ (green trace) or by heating to 140 K and recooling at 1 K min^–1^ (black trace). After recooling, both samples were scanned at a heating rate of 10 K min^–1^ to produce the calorimetry traces shown in the figure.

**Figure 3:**
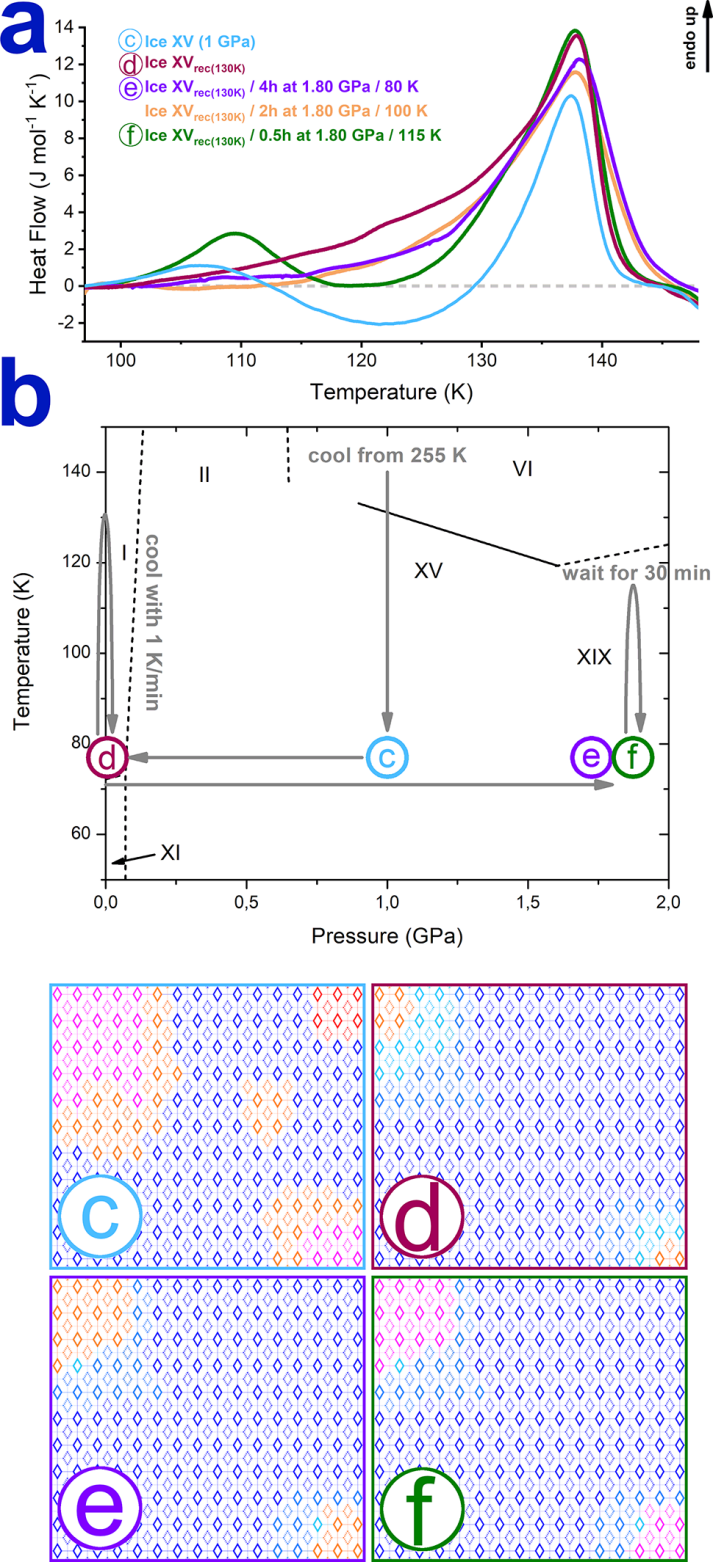
Third set of experiments. Calorimetry traces and illustration of domain structures in ice VI/XV/XIX structures prepared through different thermodynamic paths: (a) Calorimetry traces of different samples prepared through the thermodynamic paths highlighted in (b). In (b), (c) refers to an ice XV sample as prepared in ref. (3), (d) refers to ice XV recooled from 130 K at ambient pressure, (e) refers to recooled ice XV pressurized to 1.8 GPa, and (f) refers to recooled ice XV pressurized to 1.80 GPa and annealed for 30 min at 115 K. (c–f) Schematic representation of the domain structure, comprising domains of ice VI (red diamonds), ice XV (blue diamonds), ice XIX (magenta diamonds), ice VI^‡^ (orange diamonds), and ill-defined states close to ice XV (light blue diamonds). The colored, circled letters indicate the corresponding calorimetry trace in (a).

For the data in Figures 2 and 3, some of the samples mentioned above were heated and cooled a second time after the release of pressure. We refer to these samples as “recooled”. Specifically, we used an ice XIX sample (containing 5% D_2_O) cooled at 1.80 GPa as mentioned above for the data in Figure 2. The recooling process was done directly inside the calorimetry instrument at ambient pressure (see below), where the sample recovered from high pressure was cold-loaded to the crucible, heated, and cooled at 1 K min^–1^ to 77 K. For the data in Figure 3, we used an ice XV sample (containing 5% D_2_O) rapidly quenched at 1.00 GPa. After releasing the pressure, the sample was heated to 130 K at 4 K min^–1^ and cooled to 77 K at 1 K min^–1^ to obtain ice XV_rec(130 K)_.

### Sample Characterization

All of the samples were characterized using both X-ray diffraction and differential scanning calorimetry. A cylindrical sample batch recovered from the piston–cylinder equipment was 600 mg. For calorimetry and diffraction experiments, small chunks were cut from the cylinder and transferred under liquid nitrogen to the instrument. More details about the instruments and data acquisition and handling are provided in the Supporting Information.

## Results

### High Pressures

In the first set of experiments, we cooled ice VI from 250 to 77 K at pressures in the range of 0.85–2.00 GPa (as sketched in Figure 1a). After pressure release and cold loading to a differential scanning calorimeter, both at 77 K, the samples are characterized thermally upon heating. Rearrangements in the H-subnetwork are observed up to ≈148 K (Figure 1b) followed by the reconstruction of the O-atom network to ice I, which involves expansion by about 42%.^18,19^ Up to ≈148 K, ice VI itself merely produces a baseline featuring a subtle glass transition^3,20^ so that calorimetry does not allow for the quantification of the ice VI fraction. Ice XV features a single endotherm near 130 K, indicating the disordering transition to ice VI. Ice XIX, by contrast, shows a very complex thermal signature featuring two endotherms (near 100 and 130 K), and an exotherm in between. The first endotherm has been assigned to the H-disordering of ice XIX and the second one to the H-disordering of ice XV.^1,4^ The exotherm near 120 K signifies an H-ordering process: nascent ice VI (from ice XIX) orders to ice XV, which is thermodynamically more stable than ice VI at 120 K. The observation of the exotherm precludes ice XV at 77 K. This is also refuted based on our neutron diffraction data: the ice XIX sample prepared at 1.80 GPa reveals no evidence for any initial ice XV at 80 K, but ice XV slowly appears above 120 K at ambient pressure.^1^ The transient character of ice VI emerging from ice XIX leads us to mark it as a transition state, ice VI^‡^.^1,10^

While the ice XV thermogram is observed for samples prepared at 0.85 GPa, this is no longer so for 1.00 GPa samples (pink vs light blue traces in Figure 1b). A very weak first endotherm, followed by an exotherm, appears, whereas the massive second endotherm remains. This signifies the presence of a small amount of ice XIX at 77 K in the 1.00 GPa sample, but still a vast majority of ice XV. The same three signals are observed in all other traces in Figure 1b, where transformation enthalpies are collected in Figure 1c. While the first endotherm always grows with pressure, the second endotherm is the smallest at around 1.70 GPa. It shrinks up to 1.70 GPa because the amount of ice XV at 77 K decreases from 100 to 0%. It then increases again because the amount of ice XV formed during the scan near 120 K from ice VI^‡^ increases. That is, the thermograms demonstrate the competition between two ways of ice XV formation: (i) through cooling ice VI at high pressure and (ii) through heating ice XIX at ambient pressure. Consistent with this, also the area of the ice VI^‡^ exotherm is the smallest at 1.70 GPa. This good correlation between the area of the exotherm and the area of the second endotherm (compare orange and pink curves in Figure 1c) strongly enforces our argument for ice VI^‡^: if more ice XV originates from ice VI^‡^ near 120 K, releasing heat, then also more ice XV disorders near 130 K, taking up the heat.

It is also instructive to define the ratio of the two endotherm areas as a measure for the ice XV/XIX ratio at 77 K (Figure 1d). This ratio is zero in the absence of ice XIX, increases steeply from <0.1 to >1.0 as the ice XV fraction goes down, and ultimately levels off near 1.5 when ice XV is absent at 77 K. The sigmoidal shape of the curve is typical of a competition between two types of kinetics, where ice XV formation dominates at low pressure (≤0.85 GPa) and ice XIX formation at high pressure (>1.60 GPa). At 1.00 GPa, which was previously used for making “ice XV”,^3,7^ the competition already produces mixed ices containing a small amount of ice XIX domains. This is qualitatively similar for fully protiated and 95% H/5% D-samples (Figure 1d). At 1.80 GPa, all ordered domains are ice XIX, with no ice XV being present in both cases. Yet, in fully protiated samples, a lower plateau value for an ice XIX/XV peak area ratio of 1.1 is reached. This indicates that partial deuteration slows down the ice XIX → ice VI^‡^ → ice XV cascade at ambient pressure.

### Recooling at Ambient Pressure

The second set of experiments is devoted to a deeper understanding of the complex transformation sequence at ambient pressure. To this end, we recooled the 1.80 GPa ice XIX sample (green trace in Figure 1b) at 1 K min^–1^ at ambient pressure (i) from nascent ice VI^‡^ at 118 K and (ii) from stable ice VI at 140 K. At 1 bar, ice VI orders upon cooling to form ice XV,^1,3,8,10^ whereas ice XIX formation kinetics is by far too slow to be relevant. Figure 2 shows the reheating scan, where evidently the XIX → VI^‡^ endotherm is missing. Upon heating to 118 K (or beyond), ice XIX disappears, where ice XV forms upon recooling at ambient pressure. The area of the ice XV → VI endotherm significantly grows from 45 to 65 J mol^–1^ for case (i) and to 150 J mol^–1^ for case (ii). That is, some ice XV is gained upon recooling ice VI^‡^, and large amounts are produced from stable ice VI. The tripling in size compared to the pressure-cooled ice XV sample^3,9,10,21−23^ suggests a better ordering and faster kinetics at ambient pressure. Moreover, the shape of the endotherm in recooled ice XV (Figure 2) is quite different from the shape in pressure-cooled ice XV (see the pink curve in Figure 1b). First, the peak width increases from about 15 to 45 K, mainly due to fronting. Second, the endotherm features a prominent shoulder, or maybe even a second peak, just above 120 K. This hints at a range of states similar to ice XV, e.g., incompletely ordered ice XV domains slowly developing from ice VI^‡^. Each of these states is characterized through an individual onset temperature lower than the ice XV disordering temperature. This finding explains the significant differences in terms of deuteron occupation probabilities for pressure-cooled and recooled ice XV in the neutron diffraction study by Salzmann et al.^3^ That is, when referring to ice XV, it is imperative to differentiate between recooled and pressure-cooled samples. Furthermore, it is also necessary to distinguish recooled samples heated to 118 or 140 K prior to the cooling. We use the notion “ice XV_rec(118 K)_” and “ice XV_rec(140 K)_” as suggested earlier in ref. (10), where ice XV_rec(140 K)_ is much more ordered than all other variants of ice XV produced through different pathways.

### Ice XV to XIX Transition

Let us finally move to the last set of calorimetric experiments. The observation of the ice XIX → ice VI^‡^ → ice XV cascade at 1 bar gives rise to the question whether this process can be reversed. This can be answered through a series of experiments involving heating of recooled ice XV at high pressure. To this end, we have investigated the complex thermodynamic path depicted in Figure 3b step by step. In the first step, we made pressure-cooled ice XV at 1.00 GPa, which corresponds to the ice XV discovered by Salzmann et al.^3^ (circled c in Figure 3b). This ice XV was then decompressed to ambient pressure, heated to 130 K, and slowly recooled, resulting in ice XV_rec(130 K)_^10^ (circled d in Figure 3b). Next, we compressed ice XV_rec(130 K)_ to 1.80 GPa and kept it isothermally at 80 K for 4 h (circled e in Figure 3b). Two samples were additionally heated, kept isothermally, and cooled, namely at 100 K for 2 h and at 115 K for 30 min (circled f in Figure 3b). The cooling rate at 1.8 GPa to 77 K is 1 K min^–1^ to allow for a significant amount of time of domain reordering. The collected calorimetry scans of these samples in Figure 3a allow answering whether ice XV_rec(130 K)_ (circled d in Figure 3b) has converted to ice XIX at 1.80 GPa or not. Unprocessed ice XV_rec(130 K)_ (wine red trace in Figure 3a labeled with circled d) is calorimetrically identical to ice XV_rec(140 K)_ (black trace in Figure 2) within the experimental error. Our idea for the domain structures encountered in the different stages of this experiment as deduced from the calorimetry experiment is illustrated in Figure 3c–f. Pressure-cooled ice XV (Figure 3c) contains mostly ice XV domains (blue diamonds), but also domains of ice XIX (magenta diamonds) as discussed above. Furthermore, unconverted ice VI (red diamonds) and transient ice VI^‡^ (orange diamonds) are likely found in the sample. While ice VI cannot be directly assessed from the calorimetry trace, the relatively small latent heat for the ice XV endotherm speaks in favor of unconverted ice VI. Ice VI^‡^ domains are present, e.g., at the interface between the ice XIX and ice XV domains. These domains are ill-defined and may be closer to either ice XV or ice XIX. The domain structure is significantly different in recooled ice XV (Figure 3d). As alluded to above, all ice XIX domains are gone, and the sample mostly contains ice XV, with some unconverted ice VI^‡^ remaining. Ice VI^‡^ that has almost reached the final ice XV structure is represented by different shades of blue. Figure 3e shows how the domain structure is altered through compression to 1.80 GPa and keeping the sample at 1.80 GPa at 80 K for 4 h (Figure 3a, violet trace labeled with circled e). Exactly the same calorimetry trace and hence domain structure is retained by additionally heating the sample at 1.80 GPa to 100 K, keeping it there for 2 h, and cooling it back slowly to 80 K (Figure 3a, dark yellow trace). Both protocols lead to XV → VI transition enthalpies of 127 J mol^–1^, 30 J mol^–1^ smaller than that for unprocessed ice XV_rec(130 K)_. Also, the peak fronting and the width are reduced from about 45 to 35 K. This suggests that some of the less stable domains of recooled ice XV tend to disorder at 1.80 GPa, converting to ice VI^‡^. No ice XIX reappears at 80 or 100 K and 1.80 GPa. Figure 3f finally shows the domain structure of the sample after keeping ice XV at 1.80 GPa and 115 K. The most notable change in the calorimetry scan (Figure 3a, green trace labeled with circled f) is the appearance of an additional endotherm at about 100 K of 16 J mol^–1^. The XV → VI enthalpy decreases to 105 J mol^–1^, and the peak width decreases to less than 20 K. That is, about 1/3 of the ice XV domains in ice XV_rec(130 K)_ have converted to ice VI^‡^ and some ice XIX. While still far from being a complete back-transformation XV → XIX, this experiment demonstrates that the order–order transition in the triangular relation can in principle take place in both directions. Yet, about two-thirds of the ice XV domains remain unaffected. It seems that the fully developed, most stable ice XV domains are unaffected at 1.80 GPa. Only partly developed, less stable ice XV domains can be converted back to ice VI^‡^ or ice XIX. It needs to be investigated in future experiments whether all ice XV domains, including the stable ones, can be converted to ice XIX, maybe at higher pressure or after longer waiting. Higher temperatures cannot be probed because at 125 K and 1.80 GPa, the stable phase is already ice VI according to Yamane et al.^2^ so that ice XV would convert to stable ice VI, not ice XIX.

## Discussion and Conclusions

**Figure 4:**
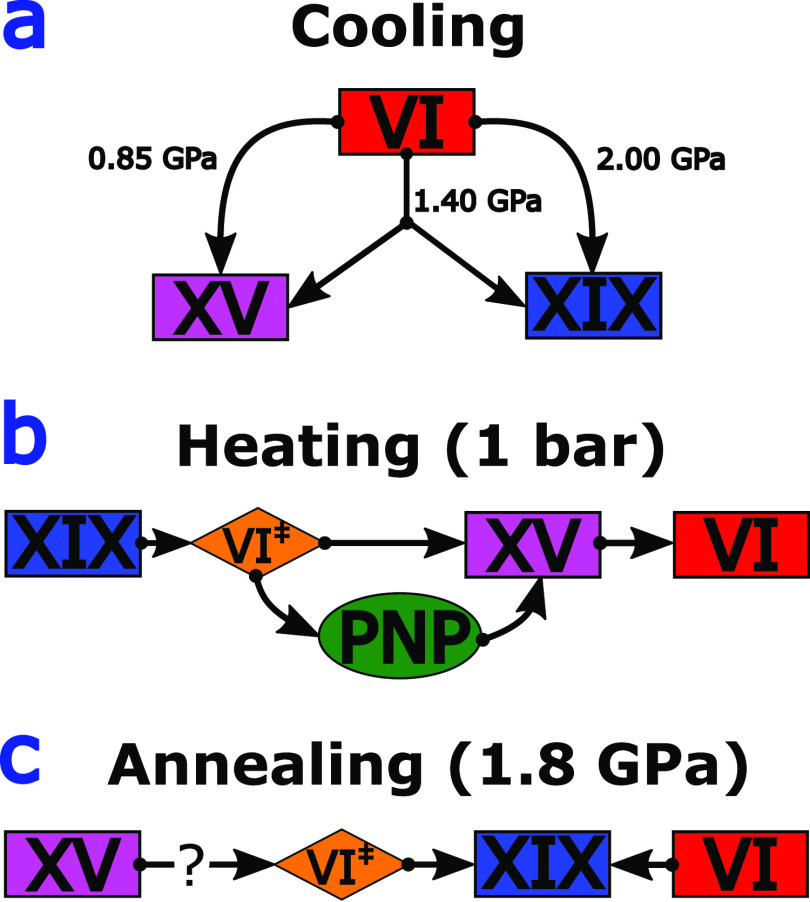
Schematic triangular relationship between ices VI, XV, and XIX as revealed from the first (a), second (b), and third (c) set of experiments outlined here. Stable phases are in rectangles, transient states in diamonds, and the possible new phase (PNP) in the ellipse.

Figure 4 summarizes the main findings from our calorimetric study on transitions in the H-atom subnetwork in H_2_O samples of ice VI topology. The ice VI topology is of high interest because it is the only one that in addition to the H-disordered parent phase features two types of H-ordered polymorphs, namely, ice XV and ice XIX. Analogously to other ice polymorphs, both of them only originate from their parent phase in the presence of point defects in their lattice, as they enhance the mobility.^1,3,4^ We introduce these point defects through the use of a dilute solution of HCl, producing a combination of ionic H_3_O^+^ defects in combination with Bjerrum-L-defects. This combination of point defects turns out to be most efficient for the ice VI → XV transformation.^7,24^ Approximately every millionth H_2_O molecule is replaced by a HCl molecule substitutionally in the ice lattice. As has been shown previously in the examples of ice V/XIII, VI/XV, and XII/XIV, this does not affect the global structure, phase behavior, or thermodynamics of the ice phase, yet enhances the mobility of the H-atoms in the ice lattice by 3–4 orders of magnitude. The case of doped ice XIX is unique, showing the most complex thermodynamics involving three stable ice phases. We here aim at resolving kinetic aspects governing the phase behavior of ice XIX based on the method of calorimetry that has helped to create our understanding of H-atom dynamics in ice phases for many decades.^3,24,25^ The ordering process also shows a huge isotope effect in deuterated samples, where deuteration slows down the sublattice dynamics tremendously. The sublattice dynamics in deuterated ice VI can be massively accelerated by adding small amounts of H_2_O.^1^ The reason for the much faster dynamics in such isotopically mixed samples is the subject of our ongoing research. In the present work, we reveal pressure as an excellent parameter for governing the kinetics of ice XV and XIX formation upon cooling protiated ice VI. The rate of ice XIX formation increases with increasing pressure, but the rate of ice XV formation decreases with increasing pressure. Below 1.00 GPa, ice XIX formation is too slow to be significant, whereas above 1.60 GPa, ice XV formation is too slow. Between 1.00 and 1.60 GPa, there is a strong competition, with the formation of both ice XV and XIX domains. Furthermore, some of the initial ice VI remains disordered throughout. This competition is sketched in Figure 4a.

Figure 4b sketches the cascade incurred upon reheating ice XIX at ambient pressure. Some of the complexity of the transition sequence comes from the fact that there is both a channel for ice XV formation and for ice XV disordering at ambient pressure. By contrast, ice XIX never forms at 1 bar but always disorders to ice VI^‡^, starting above 100 K. At 100–129 K, nascent ice VI^‡^ slowly converts to ice XV, which itself transforms into stable ice VI above 129 K. The fraction of ice VI^‡^ that was lacking time to transform into ice XV remains ice VI, whereas the fraction that had converted to ice XV turns back into ice VI. This rich behavior upon heating also impacts the recooling process at 1 bar, yielding recooled ice XV. While pressure-cooled ice XV at 0.85 GPa shows a relatively sharp endotherm, the peak width of recooled ice XV is 3 times larger. This implies an inhomogeneous domain structure of recooled ice XV, where each differently ordered ice XV domain shows a unique onset temperature of disordering. Notably, a prominent shoulder just above 120 K, visible in Figure 2, leaves room for speculation. We think that this shoulder could arise from a well-defined type of domain, different from both ice XIX and XV. In other words, we see this as a hint for a possible new phase (PNP) within the ice VI framework of O-atoms (PNP ellipse in Figure 4b). What was regarded as a disordered, deep glassy network of H-atoms by Rosu-Finsen et al.,^8,9^ thus, turns out to be a mixture of several differently ordered domains, where domain walls separate them. Two of these domain structures are known today, ice XV and XIX, and others such as the *Cc* phase^13^ might find experimental corroboration in future crystallographic work. As pointed out by the reviewers of our work, the *Cc* phase might actually be a *Pc* phase consistent with structures #25 and #38 in Supporting Information Table S3 in ref. (16).

While ice XIX is thermodynamically less stable than ice XV at ambient pressure, it is more stable at 1.80 GPa. Our attempt to convert ice XV to XIX at 1.80 GPa was partly successful. At 115 K we find calorimetric evidence that some of the recooled ice XV domains transform into ice XIX. Yet, about two-thirds of the ice XV domains are unaffected. This suggests ice XIX to be indeed more stable at higher pressures, but a rather high activation barrier for the process. Especially, fully developed ice XV domains seem to resist back-transformation into ice XIX. For this reason, we have placed a question mark in Figure 4c, leaving it open whether full back-conversion from ice XV to XIX can be achieved.

These results reveal the subtle interplay of thermodynamics and kinetics that governs the type of H-ordering and its rate of formation as a function of pressure. As such, this represents a great benchmark for any kind of potential describing H-bonded networks, especially condensed water. There are currently no potentials that accurately describe this competition between different types of H-orders to the best of our knowledge. In addition to the importance of describing the H-bond interactions in this many-body system exactly, the results are also of interest in our understanding of ice in the interior of planets and moons. Ice VI is thought to exist in the interior of many outer planets and icy moons,^26^ such as Ganymede^27,28^ or Titan,^29^ a few hundred kilometers deep inside its icy mantle. Ice VI is also known to exist in the mantle of earth, where ice VI inclusions were even discovered as diamond inclusion at the earth surface.^30,31^ Depending on dopants, pressure, and especially temperature H-ordering processes might also take place in the interior of moons or planets, where H-ordering massively alters the electrical and mechanical properties. While H-disordered ices feature high relative permittivities (ε > 100), the ordered pendants show quite low permittivities (ε < 3).^32^ Also the plastic deformation and creep behavior differ very much, depending on the type of H-bond network.^33,34^ For this reason, it is of crucial importance to know how and how fast the H-sublattice (dis)orders as a function of temperature and pressure—the key question addressed in the present work. For Ganymede and other icy Jupiter and Saturn moons in our solar system, it seems that the temperatures in the interior are probably too high to allow for H-ordering of ice VI.

## Supporting Information Available

Methodological description for powder X-ray diffraction (XRD) and differential scanning calorimetry (DSC); and XRD patterns for samples described in the main manuscript (PDF)
