# Supplementary material for: Calorimetric Investigation of Hydrogen-Atom Sublattice Transitions in the Ice VI/XV/XIX Trio
Source: J Phys Chem B. 2021 Oct 14;125(42):11777–83. doi: 10.1021/acs.jpcb.1c07508 (PMC8558864; doi:10.1021/acs.jpcb.1c07508)
Supplement: Supplementary file 1 — jp1c07508_si_001.pdf [file jp1c07508_si_001.pdf]

Supporting Information

Calorimetric Investigation of Hydrogen-Atom Sublattice Transitions in the Ice VI/XV/XIX Trio

Tobias M. Gasser[1], Alexander V. Thoeny[1], Victoria Greussing[1], Thomas Loerting[1,*]

[1]*Institute of Physical Chemistry, University of Innsbruck, 6020 Innsbruck, Austria*

e-mail: thomas.loerting@uibk.ac.at

(Dated: May 20th, 2021)

I. Characterisation of samples with differential scanning calorimetry

All batches were characterised calorimetrically using a Perkin Elmer DSC 8000 differential scanning calorimeter. To this end, about 10 – 20 mg of ice were filled into aluminium crucibles under liquid nitrogen and transferred to the precooled oven of the calorimeter. Each sample was heated to 253 K at 10 Kmin^{-1}. The resulting ice I_h [1] was cooled to 93 K and heated a second time to 313 K. The second, featureless heating run was employed as baseline for the first scan. The baseline-corrected thermograms are shifted to a heat flow of zero. To determine the exact sample mass, the area under the ice melting endotherm was used, where the peak area was converted to the latent heat of fusion and compared with the heat of fusion of H_2O, 6012 Jmol^{-1} [2].

II. Characterisation of samples with powder X-ray diffraction

For X-ray diffraction experiments chunks of about 30 – 100 mg were powdered and placed on the sample holder at cryoconditions (usually at 80 K). The X-ray patterns were recorded with a (i) Siemens D5000 diffractometer with a Cu-Ni sample holder (measurements at 77 K) using a Göbel-mirror for parallel beam geometry or with a (ii) Bruker D8 Advance powder diffractometer with a Cu sample holder equipped with a PheniX Helium Cryostat (measurements at 20 K) using a Göbel-mirror or (iii) with the Bruker D8 Advance instrument in Bragg-Brentano geometry using a motorised divergence slit. All data were collected using a Cu Kα source (0.15406 nm). The peak intensities were normalized to match at d-spacings of 0.252 nm, the position of the most intense feature in ice VI.

In general, X-ray diffraction is most sensitive to the lattice of oxygen atoms in ice samples. By contrast, the positions of H (or D) atoms barely influence the pattern. However, some small Bragg peak shifts are caused because different kinds of sublattices of H-atoms slightly displace the oxygen atoms. We reported such shifts between ice VI, XV and XIX in our earlier work [3]. Nonetheless, all ice VI, XV and XIX samples studied in the present work (and presented in Supplementary Figures 1, 2 and 3) are very similar and compared to the ice VI pattern [4]. In addition to ice VI/XV/XIX the patterns contain very small Bragg peaks related to ice Ih, which indicates that a very small amount of humidity has condensed onto the samples during transfer. That is, all the samples prepared in the present work contain the same network of oxygen atoms found in ice VI, ice XV and ice XIX. A clear distinction between different H-ordered and H-disordered polymorphs and analysis of domain ordering is difficult to make based on X-ray diffraction alone. For this reason the calorimetric data are more telling about the H-atom sublattice transitions and the main focus of the manuscript. We do

want to note, though, that especially the single Bragg peak at d-spacings of about 0.32 nm seems to split into two Bragg peaks (or develops a shoulder) for the three samples in Supplementary Figure 3. This might indicate that a different domain structure of the H-atoms prevails in repressurized and recooled samples, compared to the ice VI, XV and XIX structures described in earlier literature [3-11]. This aspect is consistent with our interpretation of the calorimetry data and needs to be investigated in future work based on neutron diffraction, which is much more sensitive to D-atom positions than X-ray diffraction.

Figure S1: Powder X-ray diffractograms of sample batches shown in Figure 1 of the main manuscript. All patterns were measured using the Siemens D5000 instrument. Grey areas mark the region of Cu and Ni sample holder Bragg peaks. Reflex positions for ice VI [4] and ice I_h [12] are indicated as tick marks as a reference. The weak Bragg peaks near 0.33 nm arise from a contamination of the sample holder itself that persists up to room temperature.

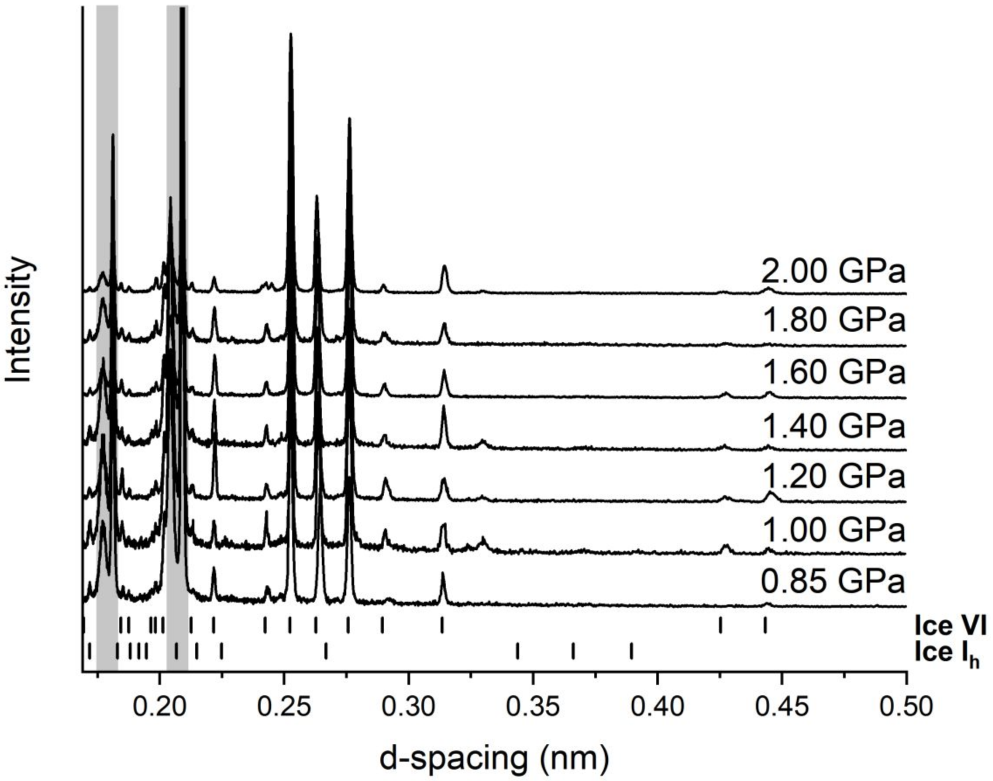

Figure S2: Powder X-ray diffractograms of the sample batch shown in Figure 2 of the main manuscript. The pattern was measured using the Siemens D5000 instrument. Grey areas mark the region of Cu and Ni sample holder Bragg peaks. Reflex positions for ice VI [4] and ice I_h [12] are indicated as tick marks as a reference.

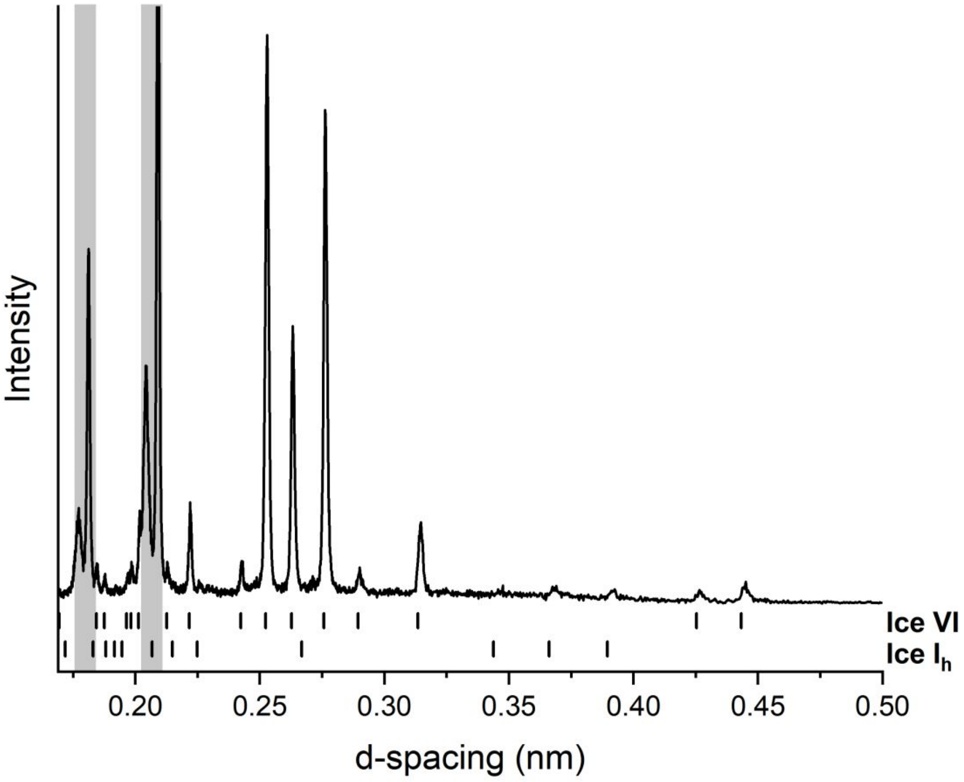

Figure S3: Powder X-ray diffractograms of sample batches shown in Figure 3 of the main manuscript. Ice XV prepared at 1.0 GPa was measured using the Siemens D5000 diffractometer. All other traces were measured using the Bruker D8 Advance powder diffractometer in Bragg-Brentano (BB) or Göbel-mirror (GM) configuration. Grey areas mark the region of Cu and Ni sample holder Bragg peaks. Reflex positions for ice VI [4] and ice I_h [12] are indicated as tick marks as a reference.

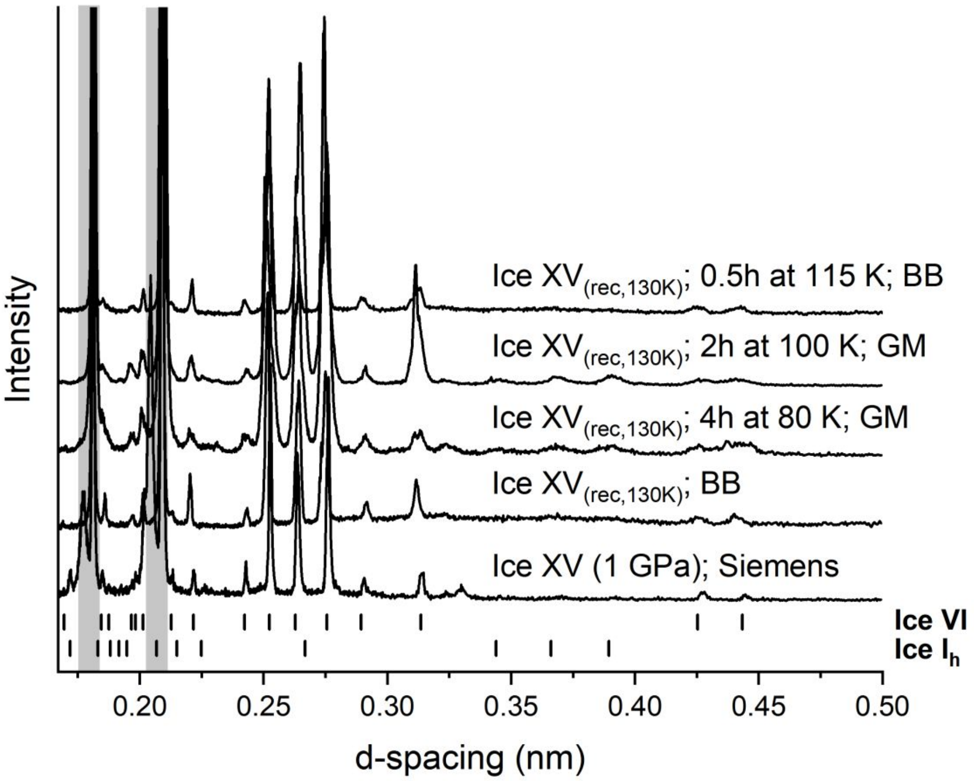

Supplemental References:

1. Handa, Y. P.; Klug, D. D.; Whalley, E., Energies of the phases of ice at low-temperature and pressure relative to ice Ih. *Can. J. Chem.* **1988,** *66* (4), 919-924.
2. Hallbrucker, A.; Mayer, E., Calorimetric Study of the Vitrified Liquid Water to Cubic Ice Phase Transition. *J. Phys. Chem.* **1987,** *91* (3), 503-505.
3. Gasser, T. M.; Thoeny, A. V.; Plaga, L. J.; Köster, K. W.; Etter, M.; Böhmer, R.; Loerting, T., Experiments indicating a second hydrogen ordered phase of ice VI. *Chem. Sci.* **2018,** *9* (18), 4224-4234.
4. Kamb, B., Structure of Ice VI. *Science* **1965,** *150* (3693), 205-209.
5. Gasser, T. M.; Thoeny, A. V.; Fortes, A. D.; Loerting, T., Structural characterization of ice XIX as the second polymorph related to ice VI. *Nat. Commun.* **2021,** *12* (1), 10.
6. Kuhs, W. F.; Finney, J. L.; Vettier, C.; Bliss, D. V., Structure and hydrogen ordering in ices VI, VII, and VIII by neutron powder diffraction. *J. Chem. Phys.* **1984,** *81* (8), 3612-3623.
7. Kuo, J. L.; Kuhs, W. F., A First Principles Study on the Structure of ice-VI: Static Distortion, Molecular Geometry, and Proton Ordering. *J. Phys. Chem. B* **2006,** *110* (8), 3697-3703.
8. Salzmann, C. G.; Radaelli, P. G.; Mayer, E.; Finney, J. L., Ice XV: A New Thermodynamically Stable Phase of Ice. *Phys. Rev. Lett.* **2009,** *103* (10), 105701.
9. Salzmann, C. G.; Slater, B.; Radaelli, P. G.; Finney, J. L.; Shephard, J. J.; Rosillo-Lopez, M.; Hindley, J., Detailed crystallographic analysis of the ice VI to ice XV hydrogen ordering phase transition. *J. Chem. Phys.* **2016,** *145* (20), 204501.
10. Rosu-Finsen, A.; Amon, A.; Armstrong, J.; Fernandez-Alonso, F.; Salzmann, C. G., Deep-Glassy Ice VI Revealed with a Combination of Neutron Spectroscopy and Diffraction. *J. Phys. Chem. Lett.* **2020,** *11* (3), 1106-1111.
11. Yamane, R.; Komatsu, K.; Gouchi, J.; Uwatoko, Y.; Machida, S.; Hattori, T.; Ito, H.; Kagi, H., Experimental evidence for the existence of a second partially-ordered phase of ice VI. *Nat. Commun.* **2021,** *12* (1), 6.
12. Fortes, A. D.; Wood, I. G.; Grigoriev, D.; Alfredsson, M.; Kipfstuhl, S.; Knight, K. S.; Smith, R. I., No evidence for large-scale proton ordering in Antarctic ice from powder neutron diffraction. *J. Chem. Phys.* **2004,** *120* (24), 11376-11379.
